# In Silico and In Vitro Study of Antioxidant Potential of Urolithins

**DOI:** 10.3390/antiox12030697

**Published:** 2023-03-11

**Authors:** Emanuela Marchese, Valentina Orlandi, Federica Turrini, Isabella Romeo, Raffaella Boggia, Stefano Alcaro, Giosuè Costa

**Affiliations:** 1 Dipartimento di Scienze della Salute, Università degli Studi “Magna Græcia” di Catanzaro, Campus “S. Venuta”, Viale Europa, 88100 Catanzaro, Italy; 2 Dipartimento di Farmacia, Università degli Studi di Genova, Viale Cembrano 4, 16148 Genova, Italy; 3 Net4Science Academic Spin-Off, Università degli Studi “Magna Græcia” di Catanzaro, Campus “S. Venuta”, Viale Europa, 88100 Catanzaro, Italy; 4 Associazione CRISEA-Centro di Ricerca e Servizi Avanzati per l’innovazione Rurale, Loc. Condoleo, 88055 Belcastro, Italy

**Keywords:** antioxidant mechanism, urolithins, DFT, kinetic constants, DPPH, FRAP, radical scavenging

## Abstract

In this work, quantum chemical calculations based on density functional theory (DFT) were performed to predict the antioxidant potential of four bioactive gut microbiota metabolites of the natural polyphenols ellagitannins (ETs) and ellagic acid (EA), also known as urolithins (UROs). In order to evaluate their ability to counter the effect of oxidative stress caused by reactive oxygen species (ROS), such as the hydroperoxyl radical (^•^OOH), different reaction mechanisms were investigated, considering water and lipid-like environments. Through our in silico results, it emerged that at physiological pH, the scavenging activity of all urolithins, except urolithin B, are higher than that of trolox and other potent antioxidants existing in nature, such as EA, α-mangostin, allicin, caffeine and melatonin. These findings were confirmed by experimental assays.

## 1. Introduction

Since ancient times, pomegranate (*Punica granatum* L.) has been a crop exploited by humankind and very appreciated by consumers due to its high-quality attributes.

In recent years, the growing interest in pomegranate is based on the benefits that its fruits have demonstrated for human health [1,2,3]. The nutraceutical properties of pomegranate are not limited to the edible part, but are shared by different parts of the fruit (i.e., peels and seeds) and of the tree (i.e., barks, buds, leaves), which in some cases contain higher quantities of biologically active compounds compared to the arils [4,5]. For this reason, in the last decade, many researchers have focused on the valorization of pomegranate processing by products using innovative and low-impact extraction techniques, i.e., ultrasound-assisted extraction, microwave-assisted extraction, supercritical fluid extraction, pressurized liquid extraction and eutectic solvent mixture, as green alternatives to conventional extraction methods [6,7,8,9,10].

Pomegranate has been recognized as a “superfood”, namely a functional product rich in bioactive compounds, such as antioxidants, minerals, vitamins and fiber (i.e., pectin). In particular, it is a rich source of ellagitannins (ETs), an important class of natural polyphenols classified as hydrolysable tannins (HTs) [1]. Structurally, ETs are esters of hexahydroxydiphenoic acid (HHDP) and gallic acid (GA) with sugar residues, predominantly β-D-glucose [11].

**Figure 1:**
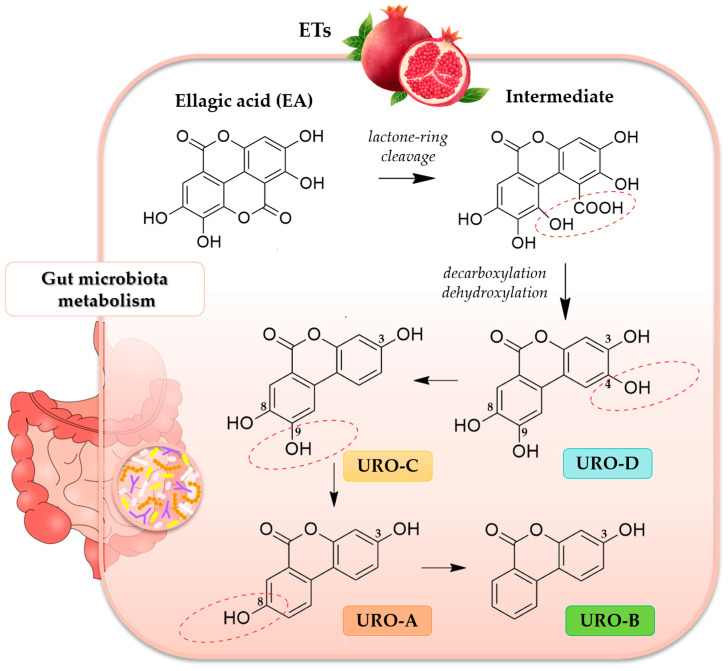
Graphical representation of gut microbiota generation of urolithins (URO-A, URO-B, URO-C, URO-D), after intake of food rich in ellagitannins (ETs), like pomegranate.

Tannins have been considered an antinutrient for a long time due to their poor bioavailability. However, ellagic acid (EA), released from ETs in the gastrointestinal tract, is partially absorbed and further metabolized by the gut microbiota to urolithins (UROs), which are regarded as the main active compounds with various bioactivity in vivo [12]. In fact, UROs, dibenzopyran-6-one derivatives with different hydroxyl substitutions, are in vivo better absorbed than EA, and they are detectable in blood and tissues [11]. The catabolic pathway of ETs and EA to UROs is very complex and includes several steps, such as lactone-ring cleavage, decarboxylation and dehydroxylation reactions, to form different urolithin intermediates, such as urolithin D (URO-D), urolithin C (URO-C), urolithin A (URO-A) and urolithin B (URO-B) [13] (Figure 1).

The EA metabolism to UROs is highly inter-individual-dependent and not always reproducible, both in terms of concentration and of pharmacological results. URO-A and URO-B are the main metabolites present in the gut and URO-A is the most biologically active compared to the other metabolites [14]. Moreover, in vivo the conjugated metabolites (such as glucuronides or sulfates) of UROs also play a determining role [15].

In the last few years, the number of research articles on UROs has exponentially increased, with significant advances in their biological effects and the production by gut microbiota. Three different urolithins metabotypes (UMs), namely ellagitannin-metabolizing phenotypes, have been described: UM 0 (no UROs producers), UM A (URO-A producers) and UM B (distinctively, URO-A, isourolithin A, and/or URO-B producers), together with a strictly age-dependent UM [16].

UROs exhibit various biological activities, including anti-inflammatory, antimicrobial, anticancer, cardioprotective, neuroprotective and antioxidant activities [13]. Their antioxidant effects are due to their ability to reduce the free radicals, and specifically, the intracellular reactive oxygen species (ROS); these metabolites also inhibit lipid peroxidation in certain cell types [17,18]. The full understanding of the chemistry involved in UROs antioxidant protection could be crucial to identify powerful strategies to reduce oxidative stress. To accomplish this, computational approaches aid in the identification and elucidation of the potential chemical routes related to the antioxidant activity, taking into account the environment’s factors and the chemical nature of the generated free radicals. In particular, quantum mechanics methods offer a universal and quantitative advance of predicting the free radical scavenging activity of a wide range of chemical entities. Therefore, in this work we have performed a theoretical investigation of the antioxidant activity of UROs by applying a density functional theory (DFT)-based in silico protocol successfully reported in previous studies [19,20,21,22]. Considering the most frequently mentioned mechanisms [23], hydrogen atom transfer (HAT) and single electron transfer (SET) have been considered in both aqueous and lipid environments. The general reaction mechanisms are:

$$\mathrm{HAT}:\;H_{n}A+R^{•}\to [H_{n-1}{A]}^{•}+HR$$

$$\mathrm{SET}:\;H_{n}A+R^{•}\to H_{n}A^{+•}+R^{-}$$

in which the chosen free radical (*R^•^*) was the hydroperoxyl radical (^•^OOH) because its half-life allows the best interception by chemical scavengers [24,25]. The quantum mechanics-based test for overall free radical scavenging activity (QM-ORSA) protocol [26] has been successfully employed to predict kinetic data for these reactions in solution. Furthermore, two in vitro experimental assays, such as the 2,2-diphenyl-1-picrylhydrazyl (DPPH) free radical scavenging method and the ferric reducing antioxidant power (FRAP), have been carried out on all studied UROs species.

## 2. Materials and Methods

### 2.1. Computational Methods

All computational studies were performed with Jaguar [27] and Jaguar pKa [28] programs, implemented in Schrödinger Suite 2022-1 [29]. The structures of UROs, such as URO-A, URO-B, URO-C and URO-D, used for the DFT studies were downloaded in SDF format from the repository of PubChem [30]. The geometries of all the investigated species were fully optimized employing the hybrid M05-2X exchange-correlation functional [31] coupled with the extended 6-311++G(d,p) basis set. Vibrational frequencies were obtained at the same level of theory. Unrestricted calculations were used for open shell systems.

The Poisson-Boltzmann Finite element (PBF) [32,33] was chosen as continuum solvation model, where the Poisson-Boltzmann (PB) equation is computed as long as the electric potential on the molecular surface reaches equilibrium, taking into account the contributions from the solvent and the solute. Pentyl ethanoate (PE) and water solutions were explored to mimic lipid and aqueous environments, respectively. Frequency calculations by means of the standard search method allowed us to verify if the obtained geometries were transition states TSs (1 imaginary frequency) and connecting local minima (0 imaginary frequency). Furthermore, intrinsic reaction coordinate (IRC) calculations were performed to ensure if TSs were correctly located. The zero-point energy corrections at 298.15 K were included in the calculation of relative energies.

For each investigated reaction mechanism, such as HAT and SET, the standard Gibbs free energy (Δ*G*°) at standard conditions (*T* = 298.15 K and P° = 1 bar) was calculated as the difference between the Gibbs free energies of products and reactants. For the kinetic studies, only exergonic reactions (Δ*G*° < 0) were considered, thus calculating activation Gibbs free energies (Δ*G*^‡^) as the differences between the Gibbs free energies of TS and reactants.

The quantum mechanics-based test for overall free radical scavenging activity (QM-ORSA) protocol [26] was applied to compute the thermal rate constants (*k*) of the reactions between UROs and hydroperoxyl radical ^•^OOH. Rate constants were determined by applying the conventional transition state theory (TST) at the 1 M standard state by using Equation (1) [34].

(1) $$k=\kappa \sigma \frac{k_{B}T}{h}e^{-\frac{{∆G}^{‡}}{RT}}$$

where κ is the tunneling correction; *σ* is the reaction path degeneracy, which considers the existence of different but equivalent reaction paths; *k*_B_ is the Boltzmann constant; *T* is the absolute temperature; *h* is the Planck constant and *R* is the ideal gas constant.

For the HAT reaction mechanism, the tunneling correction (κ) was calculated by using the Eckart method [35,36], in which information along the minimum energy path (MEP), in particular, at the stationary points (reactants, transition state and product are required). For SET mechanism, the barrier of reaction was computed using the Marcus theory [37,38], as:

(2) $${\Delta G}^{‡}=\frac{\lambda }{4}{\left(1+\frac{\Delta G}{\lambda }\right)}^{2}$$

where *λ*, expressed in kcal/mol, is a nuclear reorganization term.

Total rate constants (*k_tot_*) for each combination of mechanism and reaction site were calculated as the sums of the rate constants. In addition, branching ratios (Γ*_i_*) were calculated by using Equation (3):

(3) $$\Gamma_{i}=\frac{100k_{i}}{k_{tot}}$$

Corrected total rate constants (*fk_tot_*) for each acid-base species (*i*), weighted by their mole fractions *f_i_* at 7.4 pH, as shown in Equation (4):

(4) $$fk_{tot}=f_{i}k_{tot}$$

Overall rate constants (*k_overall_*) were computed as the sum of the corrected total rate constants for all mechanisms and reaction sites, as shown in Equation (5):

(5) $$k_{overal}=\sum_{i=1}^{n}{fk}_{tot}$$

For apparent rate constants (*k_app_*), close to the diffusion limit, the Collins–Kimball theory [39] was used, as reported in Equation (6):

(6) $$k_{app}=\frac{k_{D}k}{k_{D}+k}$$

where *k_D_* represents the steady-state rate constant for an irreversible bimolecular reaction diffusion-controlled.

### 2.2. Chemicals

All chemicals and reagents were of analytical grade. URO-A, URO-B, URO-C, URO-D, trolox, 2,2-diphenyl-1-picrylhydrazyl (DPPH) and methanol were purchased by Sigma-Aldrich (Steinheim, Germany). The colorimetric FRAP (Ferric Reducing Antioxidant Power) assay kit (ab234626) was purchased from Abcam© (Abcam, Caliph, Ml, Cambridge, UK).

### 2.3. DPPH Radical Assay

The antioxidant activity was evaluated in microplates using the purple free radical 2,2-diphenyl-1-picrylhydrazyl (DPPH^•^) radical assay following the protocol proposed by Casedas et al. [40], with slight modifications.

Metanolic solutions of EA, URO-A, URO-B, URO-C, URO-D at different concentrations (from 100 mg/mL to 1 mg/mL) were tested. In total, 150 mL of the DPPH methanolic stock solution (10^−4^ M) was added together with 150 mL of each sample methanolic solution. Methanolic solutions of trolox at different concentrations (100 mg/mL to 10 mg/mL) were used as standards. The plate was incubated for 30 min under dark conditions and then measured at 515 nm. The Inhibition Capacity (IC) was calculated by the following formula:

IC%= [(A_0_ − A_1_)/A_0_] ∗ 100

where A_0_ is the absorption of control and A_1_ is the absorption of the tested extract solution. The calculation of IC_50_ was performed using a regression line (Y = AX + B) drawn from two points enclosing the 50% inhibition ratio [41]. The sample concentration (X) was calculated by substituting the Y with the value 50 in the regression equation.

As suggested by Xiao et al. [41], the trolox equivalent antioxidant capacity (TEAC) was calculated as a ratio between the IC_50_ of trolox and the IC_50_ of the substance of interest (expressed in the same measurement unit).

### 2.4. FRAP Assay

A FRAP commercial assay kit has been used to spectrophotometrically evaluate the total antioxidant activity. This assay measures the antioxidant potential in samples through the reduction of ferric iron (Fe III) to ferrous iron (Fe II) by antioxidant compound present in the analyzed samples. The reduction of Fe III leads to the formation of an intense blue color, after an incubation time in the dark at 37 °C for 60 min, and having an absorption maximum at 594 nm. Methanolic solutions of UROs (URO-A, B, C and D) with different concentrations (from 50 mg/mL to 1 mg/mL) have been tested.

The antioxidant capacity was calculated using a Fe II standard curve, and the results were expressed as Fe II equivalents (mM).

## 3. Results

### 3.1. In Silico Thermochemical Viability of UROs

**Figure 2:**
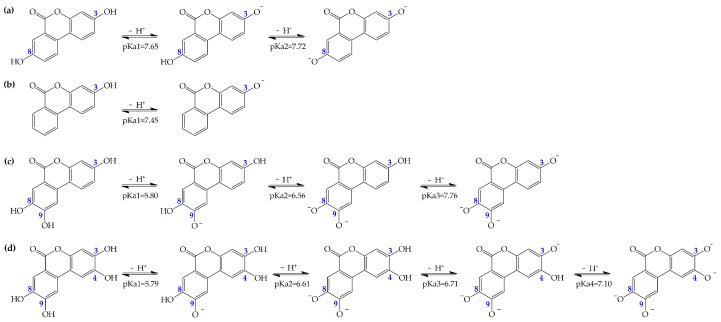
pKa values of the relative deprotonation paths of (**a**) URO-A, (**b**) URO-B, (**c**) URO-C and (**d**) URO-D at physiological pH.

Depending on the capability to offer protection against ROS, a compound can be a specific or versatile antioxidant. Also based on their acid-base equilibria, the antioxidants could affect both reactivity and membrane permeability. For this reason, our preliminary calculations are devoted to the prediction of the acid dissociation constants (pKas) of all the investigated compounds in aqueous environment at physiological pH (Figure S1). All calculations are performed by means of Jaguar pKa workflow calculations [42]. For the URO-B, a single deprotonation site is available and its predicted pka is 7.45. After knowing the pKas of each of the UROs, the deprotonation routes of the species with multiple sites are elucidated (Figure 2).

Our results indicate that the first deprotonation site in URO-A is the OH in the C3 position, followed by OH at C8. On the contrary, in URO-C and D, the sequence of deprotonation starts from the OH site in the C9 position, while the second and third ones involve sites C8 and C3, respectively. Subsequently, we quantify the relative molar fractions (*ƒ*) at pH 7.4. At pH 7.4, the neutral and dissociated forms are distributed almost equally for URO-A and URO-B, while the neutral forms are negligible in favor of the dissociated forms that are more dominant for URO-C and D (Table 1).

**Scheme 1:**
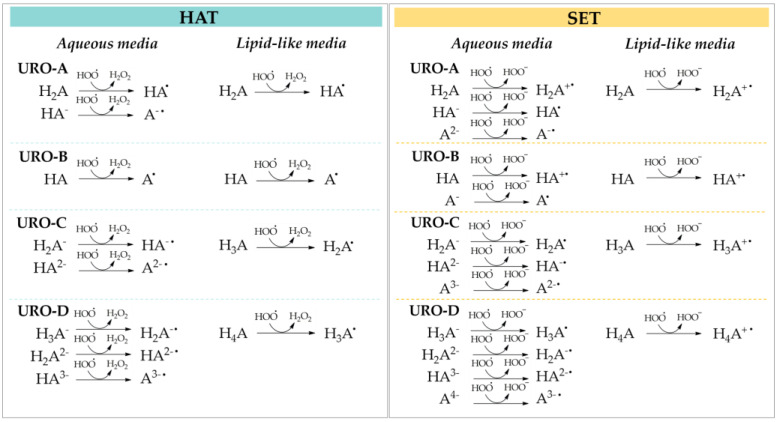
Schematic representation of the considered mechanisms for UROs and their acid–base forms: HAT, hydrogen atom transfer, SET and single-electron transfer.

Accordingly, for the thermochemical and kinetic studies in the aqueous solution, only neutral and acid-base species with *f* ≥ 0.01 (more than 1%) are considered. This is because the multifunctional antioxidants are expected to enter the cells by passively crossing biological membranes [43]. On the contrary, in lipid-like media, which does not promote the necessary solvation to stabilize the ionic species, the neutral forms are prevalent. The two investigated antioxidant mechanisms against the ^•^OOH radical are summarized in Scheme 1.

The thermochemical viability of all considered reaction paths are evaluated, in terms of their standard Gibbs free energies of reaction (Δ*G*°), in water and PE for HAT and SET mechanisms; they are summarized in Table 2 and Table 3, respectively.

Concerning the HAT mechanism in aqueous solution, both neutral and anionic species of URO-A show favorable Gibbs free energies with similar values (from −9.72 to −10.74 kcal/mol) for all involved sites. On the contrary, the ^•^OOH attack on the C3 site of URO-B results in endergonic by 19.19 kcal/mol. For URO-C, the Gibbs energy for the reaction at site 3 of the di-anionic form is −12.78 kcal/mol. About URO-D, among the most abundant dissociated forms, the di-anionic and tri-anionic ones possess a favorable Gibbs free energy, with values equal to −15.26 kcal/mol and −16.00 kcal/mol, for sites 3 and 4 of H_2_A^2−^, respectively, and −13.63 kcal/mol for the C4 site of HA^3−^.

In PE solvent, where only the neutral species are present, the HAT mechanism is favored for all UROs with the exception of site 3 of URO-D. In detail, the lowest Δ*G* values are obtained for site 4 of URO-D, followed by the comparable values computed for all sites of URO-C (from −9.32 to −9.82 kcal/mol). The HAT mechanism for URO-B is exergonic with a calculated Gibbs free energy of −6.77 kcal/mol, in contrast to its behavior in the aqueous media.

**Figure 3:**
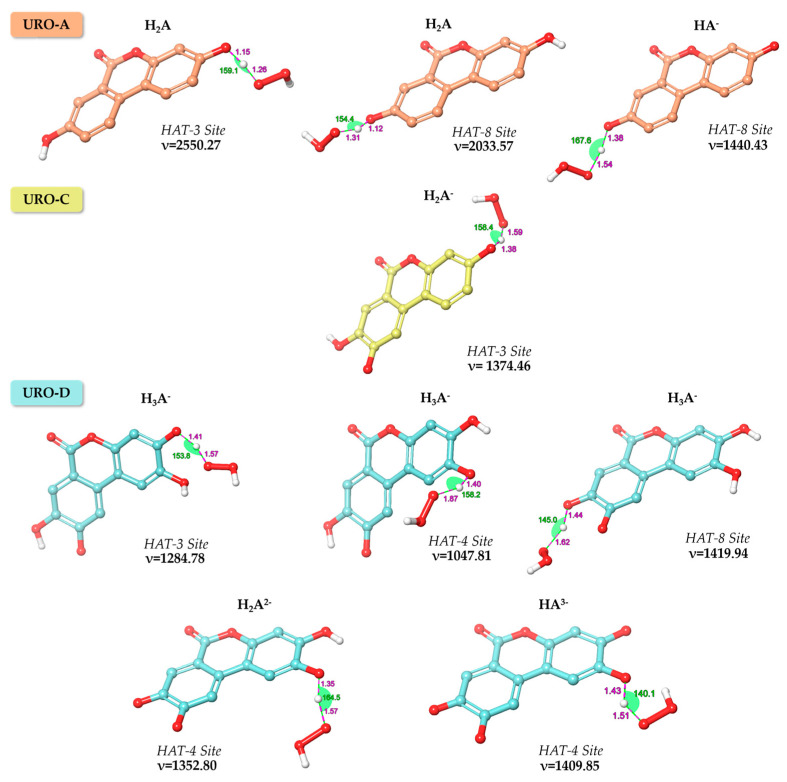
Geometries of the transition states of URO-A, URO-C and URO-D obtained for the HAT reaction pathways in water solution. Bond lengths (purple), angles (green) and imaginary frequencies (*ν*) are reported in Å, degrees and cm^−1^, respectively.

The activation energies Δ*G*^‡^ at 298.15 K, expressed in kcal/mol, are calculated only for the thermodynamically favorable reaction paths. The optimized geometries of the intercepted TSs obtained in water for the exergonic HAT processes for URO-A, URO-C and URO-D and their relative imaginary frequencies are shown in Figure 3.

As displayed in Table 2, the smaller barriers in terms of Δ*G*^‡^ are found for the di-anionic and tri-anionic forms of URO-D. Despite all the attempts made—for only the mono-anionic form of URO-D—it has not been possible to locate the TS corresponding to HAT reaction at site 3. Additionally, performing the relaxed scan obtained by reducing the H---OOH distance, the TS failed to find due to rapid H transfer to the product. For all these reasons, the reaction at site 3 for H_2_A^2−^ of URO-D is barrierless and strictly diffusion-controlled. In PE, the calculated activation energies take values in the range of about 19 to 33 kcal/mol, and the related TSs are shown in Figure S2.

As featured in Table 3, the investigated SET processes in aqueous media are characterized by exergonic energy values only for the mono-anionic forms of URO-A and URO-C (−10.83 and −7.70 kcal/mol), for the neutral form of URO-B (−5.03 kcal/mol), and even more for the mono-, tri- and tetra-anionic species of URO-D (−25.04, −16.73 and −22.20 kcal/mol) (Table S1). On the contrary, largely endergonic energies are reported in PE medium for all UROs with Δ*G*° values more than 45 kcal/mol, since this environment does not provide the necessary solvation of the intermediate ionic species produced in these reactions.

### 3.2. Kinetics (Rate Constant) Studies of UROs

In order to provide further insights into the antioxidant capability of the four UROs, we calculate the thermal kinetic constants using the thermochemical data collected in Table 2 and Table 3 and applying QM-ORSA computational protocol [26]. Therefore, for each acid base species, the individual apparent rate constants (*k_app_*) in aqueous solution for the exergonic mechanisms are summarized in Table 4. The kinetic investigations in lipidic media are depicted in Table S2.

The obtained rate constants show that SET mechanism is the faster process for all studied UROs. Particularly, for URO-A and C, the estimated rate constants are comparable, with a value of 5.63 × 10^7^ and 4.00 × 10^7^ M^−1^ s^−1^, respectively. The acid-base species of URO-D report more favored *k_app_* values equal to 7.53 × 10^9^ for H_3_A^−^, 7.90 × 10^9^ for HA^3−^ and 7.48 × 10^9^ for A^4−^. Moreover, the rate constants associated with the HAT mechanism for the di- and tri-anionic forms of URO-D are also feasible, with *k_app_* values of 2.79 × 10^9^ for both involved sites in H_2_A^2−^ and 5.64 ×10^8^ in HA^3−^.

The derived sum (*k_to_*_t_) of the apparent rate constants of all the corresponding reaction pathways for each chemical entity are reported in Table 5. Taking into account the contribution of each acid-base species at physiological pH, based on the estimated molar fractions (*f*), the rate coefficients (*fk_tot_*) are corrected. Finally, by summing-up the *fK_tot_* of all species, we found that the overall rate constant (*k_overall_*) of URO-D is higher than other UROs.

### 3.3. DPPH Radical Assay

As suggested by Xiao et al. [41], the radical scavenging activity was expressed as IC_50_, calculated by a linear regression between two points enclosing the 50% inhibition ratio allowing to approximate the trend as linear.

The results of the DPPH radical assay show, as reported in the literature, that URO-B does not have antioxidant activity, as all the methylated urolithins [44]. In contrast, the higher antioxidant activity is shown by URO-D (underlined by the lowest IC_50_ of 2.1 µg/mL) and URO-C (that shows its IC_50_ = 3.3 µg/mL). A significantly lower antioxidant power is found for URO-A, with the IC_50_ = 35.5 µg/mL, one order of magnitude higher than URO-C and URO-D. The IC_50_ and their confidence interval calculated using the inner hyperbole, since IC_50_ is a theoretical value, is reported below in Table 6. Regression data are reported in Figure S3.

The higher TEAC (Table 6) is reported for URO-D, followed by URO-C and then URO-A, where the higher TEAC means the higher DPPH scavenging activity. Since for the TEAC calculation the value of IC_50_ is required, it is impossible to calculate the TEAC for URO-B, whose antioxidant activity was not quantified by DPPH assay.

### 3.4. FRAP Assay

**Figure 4:**
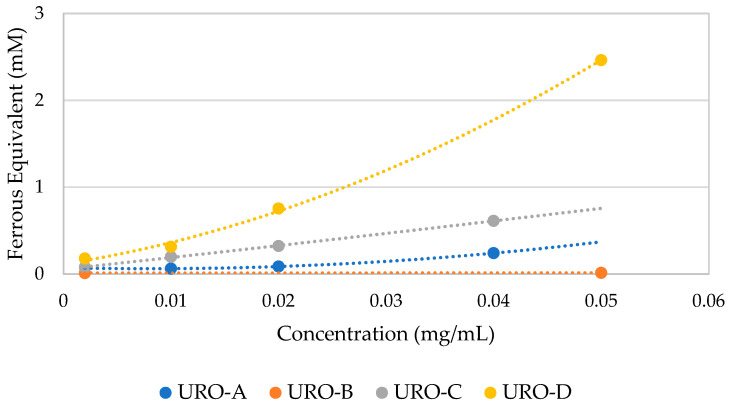
Results expressed in mM of ferrous equivalents of different concentrations (mg/mL) of URO-A, URO-B, URO-C and URO-D.

The results of FRAP assay are expressed as ferrous equivalents (mM) using a calibration curve obtained through the ferrous standard solutions included in the commercial Kit [45]. As shown in Figure 4, the ferrous equivalents (mM) are higher for URO-D, followed by URO-C and then URO-A comparing these compounds at the same concentration (mg/mL).

URO-D is confirmed as the most active antioxidant compound followed by URO-C and URO-A, while URO-B does not show any antioxidant activity.

## 4. Discussion

Starting from the knowledge of the free radical scavenging activity of EA [22,46,47] to which their beneficial effects on human health is linked [48], we conceived to analyze the antioxidant protection of its secondary metabolites, such as UROs, by in silico and in vitro investigations. As is known, a substance can be defined as an “antioxidant” if, at low concentrations, it delays or prevents the oxidation of a substrate [49]. This phenomenon may be mediated by various reaction pathways, such as the hydrogen atom transfer (HAT) and single electron transfer (SET) mechanisms. It is also necessary to consider environmental factors, pH and ionic strength of tested molecules. For this reason, an initial estimation of relative abundance of all acid-base species of UROs at physiological pH has been conducted. Analyzing our results, the antioxidant power is expected to be driven by the neutral form for URO-A, the di-anionic form for URO-C and the totally dissociated form for URO-D. By the thermochemical and kinetics calculations, it is noticeable that URO-D (*k_overall_* = 7.66 × 10^9^ M^−1^ s^−1^) is more efficient for scavenging ^•^OOH than other UROs in aqueous solution. Clearly, for the most abundant species A^4−^, SET mechanism becomes the more feasible antioxidant process with a total rate constant equal to 7.48 × 10^9^ M^−1^ s^−1^. Nevertheless, the HAT mechanism also contributes to defining the URO-D antioxidant profile, but its mono-, di- and tri-anionic species are present in small amounts. While in URO-D both the analyzed channels of reaction are possible for URO-A and URO-C, only the SET reaction is preferred, with similar velocities. To better estimate the antioxidant potential of the investigated UROs, we can cross-reference our kinetic results with those related to known antioxidants, using the same protocol. In aqueous environment, URO-A, URO-C and URO-D result to be more efficient as ^•^OOH scavengers if compared to the reference antioxidant trolox (8.96 × 10^4^ M^−1^ s^−1^) [50]. In addition, they are also more efficient than EA (1.57 × 10^5^ M^−1^ s^−1^) and other natural compounds widespread in nature, such as α-mangostin, allicin, caffeine and melatonin [46,51].

Ultimately, in vitro antioxidant capacity of UROs has been assessed through DPPH assay, which acts as mixed mode test (HAT/SET), and by FRAP assay, a SET mode test. Such experimental assays have confirmed the predicted ROS scavenging activity associated with URO-A, URO-C and URO-D, underlining that the last one shows the most antioxidant power.

## 5. Conclusions

The investigation of the potential antioxidant activity of the EA metabolites toward ^•^OOH has been conducted by using in silico and in vitro approaches. Among the analyzed UROs, URO-D is a powerful antioxidant in aqueous solution. URO-A and URO-C are less efficient than URO-D, but can also be considered good antioxidants by a comparison with the trend of antioxidant activity of trolox and other compounds naturally occurring. Instead, no antioxidant activity has been predicted for URO-B. Our computed thermochemical and kinetic data have been confirmed by experimental assays, thus corroborating that the antioxidant action is fulfilled via SET mechanism.

## Figures and Tables

**Table 1:** Molar fractions (*f*) of the acid–base species of URO A-D at pH 7.4.

URO-A		URO-B		URO-C		URO-D	
Species	*f*	Species	*f*	Species	*f*	Species	*f*
H_2_A	0.546	HA	0.529	H_3_A	0.002	H_4_A	0.000
HA^−^	0.307	A^−^	0.471	H_2_A^−^	0.085	H_3_A^−^	0.010
A^2−^	0.147			HA^2−^	0.589	H_2_A^2−^	0.063
				A^3−^	0.324	HA^3−^	0.309
						A^4−^	0.617

**Table 2:** Standard Gibbs free energies of reaction (Δ*G*°) and activation (Δ*G*^‡^), expressed in kcal/mol, at 298.15 K, in aqueous and lipidic media involved in the HAT mechanism. Energy values calculated in pentyl ethanoate (PE) are reported in parenthesis.

HAT.				
	Specie	Site	Δ*G*°	Δ*G*^‡^
**URO-A**	H_2_A	*3*	−9.77 (−7.71)	30.50 (32.66)
		*8*	−9.72 (−7.78)	29.99 (31.83)
	HA^−^	*8*	−10.74	16.95
**URO-B**	HA	*3*	19.19 (−6.77)	(32.20)
**URO-C**	H_3_A	*3*	(−9.82)	(33.27)
		*8*	(−9.32)	(34.68)
		*9*	(−9.56)	(31.62)
	H_2_A^−^	*3*	−12.78	30.59
		*8*	22.54	-
	HA^2−^	*3*	16.75	-
**URO-D**	H_4_A	*3*	(7.49)	-
		*4*	(−10.47)	(19.36)
		*8*	(−4.66)	(24.01)
		*9*	(−5.70)	(24.34)
	H_3_A^−^	*3*	−0.21	44.03
		*4*	−1.75	46.07
		*8*	−4.02	30.57
	H_2_A^2−^	*3*	−15.26	-
		*4*	−16.00	0.64
	HA^3−^	*4*	−13.63	7.27

**Table 3:** Standard Gibbs free energies of reaction (Δ*G*°) and activation (Δ*G*^‡^), expressed in kcal/mol, at 298.15 K, in aqueous and lipidic media involved in the SET mechanism. Energy values calculated in pentyl ethanoate (PE) are reported in parenthesis.

SET			
	Specie	Δ*G*°	Δ*G*^‡^
**URO-A**	H_2_A	72.08 (67.39)	-
	HA^−^	−10.83	5.47
	A^2−^	21.64	-
**URO-B**	HA	−5.03 (68.86)	21.23
	A^−^	23.41	-
**URO-C**	H_3_A	(62.05)	-
	H_2_A^−^	−7.70	7.08
	HA^2−^	19.01	-
	A^3−^	14.94	-
**URO-D**	H_4_A	(45.37)	-
	H_3_A^−^	−25.04	2.20
	H_2_A^2−^	15.89	-
	HA^3−^	−16.73	0.40
	A^4−^	−22.20	2.28

**Table 4:** Rate constants *k_app_*, expressed in M^−1^ s^−1^, in aqueous media and branching ratios (Γ) computed at M05-2X level of theory at 298.15 K.

	Specie	Site	Mechanism	*k_app_*	Γ (%)
**URO-A**	H_2_A	*3*	HAT	2.95 × 10^−4^	93.01
	H_2_A	*8*	HAT	2.21 × 10^−5^	6.99
	HA^−^	*3*	HAT	4.20 × 10^2^	∼0.00
	HA^−^	*-*	SET	5.63 × 10^7^	100.00
**URO-B**	HA	*-*	SET	1.71 × 10^−3^	100.00
**URO-C**	H_2_A^−^	*3*	HAT	2.22 × 10^−14^	∼0.00
	H_2_A^−^	*-*	SET	4.00 × 10^7^	100.00
**URO-D**	H_3_A^−^	*3*	HAT	8.91 × 10^−18^	∼0.00
	H_3_A^−^	*4*	HAT	1.01 × 10^−19^	∼0.00
	H_3_A^−^	*8*	HAT	1.08 × 10^−7^	∼0.00
	H_3_A^−^	*-*	SET	7.53 × 10^9^	100.00
	H_2_A^2−^	*3*	HAT	2.79 × 10^9^	50.00
	H_2_A^2−^	*4*	HAT	2.79 × 10^9^	50.00
	HA^3−^	*4*	HAT	5.64 ×10^8^	6.66
	HA^3−^	*-*	SET	7.90 × 10^9^	93.34
	A^4−^	*-*	SET	7.48 × 10^9^	100.00

**Table 5:** Molar fractions (*f*), total rate constants (*k_tot_*, M^−1^ s^−1^), corrected-by-fraction total rate coefficients (*fk_tot_*, M^−1^ s^−1^) and overall rate constants (*k_overall_* M^−1^ s^−1^) at 298.15 K, in aqueous solution at pH 7.4.

	Specie	*f*	*k*_tot_	*fk_tot_*	*k_overall_*
**URO-A**	H_2_A	0.546	3.17 × 10^−4^	1.73 × 10^−4^	1.73 × 10^7^
	HA^−^	0.307	5.63 × 10^7^	1.73 × 10^7^	
**URO-B**	A^−^	0.471	1.71 × 10^−3^	9.05 × 10^−4^	9.05 × 10^−4^
**URO-C**	H_2_A^−^	0.085	4.00 × 10^7^	3.40 × 10^6^	3.40 × 10^6^
**URO-D**	H_3_A^−^	0.010	7.53 × 10^9^	7.53 × 10^7^	7.66 × 10^9^
	H_2_A^2−^	0.063	5.59 × 10^9^	3.52 × 10^8^	
	HA^3−^	0.309	9.46 × 10^9^	2.62 × 10^9^	
	A^4−^	0.617	7.48 × 10^9^	4.62 × 10^9^	

**Table 6:** Confidence interval and TEAC of URO-A, URO-C and URO-D.

	IC_50_ (*p* = 95%)	TEAC
URO-A	33.1 < IC_50_ < 38.8	0.16
URO-C	3.2 < IC_50_ < 3.4	1.81
URO-D	1.9 < IC_50_ < 2.2	2.84

## Data Availability

The data presented in this study are available on request from the corresponding author.

## Supplementary Materials

The following supporting information can be downloaded at: https://www.mdpi.com/article/10.3390/antiox12030697/s1, Figure S1: Distribution diagrams of UROs as function of pH; Figure S2: Geometries of the transition states of URO-A, URO-B, URO-C and URO-D obtained for the HAT reaction pathways pentyl ethanoate (PE). Bond lengths (purple), angles (green) and imaginary frequencies (ν) are reported in Å, degrees and cm^−1^, respectively; Table S1: Nuclear organization term (λ) for the calculation of the barrier of reaction in SET mechanism, computed using the Marcus theory; Table S2: Rate Constants (*k_app_*), expressed in M^−1^ s^−1^, in pentyl ethanoate and branching ratios (Γ) computed at M05-2X level of theory at 298.15 K; Figure S3: Regression lines for the calculation of the IC_50_ values; Figure S4: (a) Calibration curve of FRAP assay; (b) the microplate containing standards and samples at different concentrations.
