# Supplementary material for: In Silico and In Vitro Study of Antioxidant Potential of Urolithins
Source: Antioxidants (Basel). 2023 Mar 11;12(3):697. doi: 10.3390/antiox12030697 (PMC10045577; doi:10.3390/antiox12030697)
Supplement: Supplementary file 1 [file antioxidants-12-00697-s001.zip › antioxidants-2249760-supplementary.pdf]

Supporting Information

In Silico and In Vitro Study of Antioxidant Potential of Urolithins

Emanuela Marchese [1], Valentina Orlandi [2], Federica Turrini [2], Isabella Romeo [1,3,*], Raffaella Boggia [2,*], Stefano Alcaro [1,3,4] and Giosuè Costa [1,3]

[1] Dipartimento di Scienze della Salute, Università degli Studi "Magna Græcia" di Catanzaro, Campus "S. Venuta", Viale Europa, 88100 Catanzaro, Italy
[2] Dipartimento di Farmacia, Università degli Studi di Genova, Viale Cembrano 4, 16148 Genova, Italy
[3] Net4Science Academic Spin-Off, Università degli Studi "Magna Græcia" di Catanzaro, Campus "S. Venuta", Viale Europa, 88100 Catanzaro, Italy
[4] Associazione CRISEA-Centro di Ricerca e Servizi Avanzati per l'innovazione Rurale, Loc. Condoleo, 88055 Belcastro, Italy
* Correspondence: isabella.romeo@unicz.it (I.R.); raffaella.boggia@unige.it (R.B.)

Contents

Figure S1. Distribution diagrams of UROs as function of pH. 2

Figure S2. Geometries of the transition states of URO-A, URO-B, URO-C and URO-D obtained for the HAT reaction pathways in pentyl ethanoate (PE). Bond lengths (purple), angles (green) and imaginary frequencies (ν) are reported in Å, degrees and cm^{-1}, respectively. 3

Table S1. Nuclear organization term (λ) for the calculation of the barrier of reaction in SET mechanism, computed using the Marcus theory. 4

Table S2. Rate Constants (k_{app}), expressed in M^{-1} s^{-1}, in pentyl ethanoate and branching ratios (Γ) computed at M05-2X level of theory at 298.15 K. 5

Figure S3. Regression lines for the calculation of the IC50 values. 6

Figure S4. a) Calibration curve of FRAP assay; **b)** The microplate containing standards and samples at different concentrations. 7

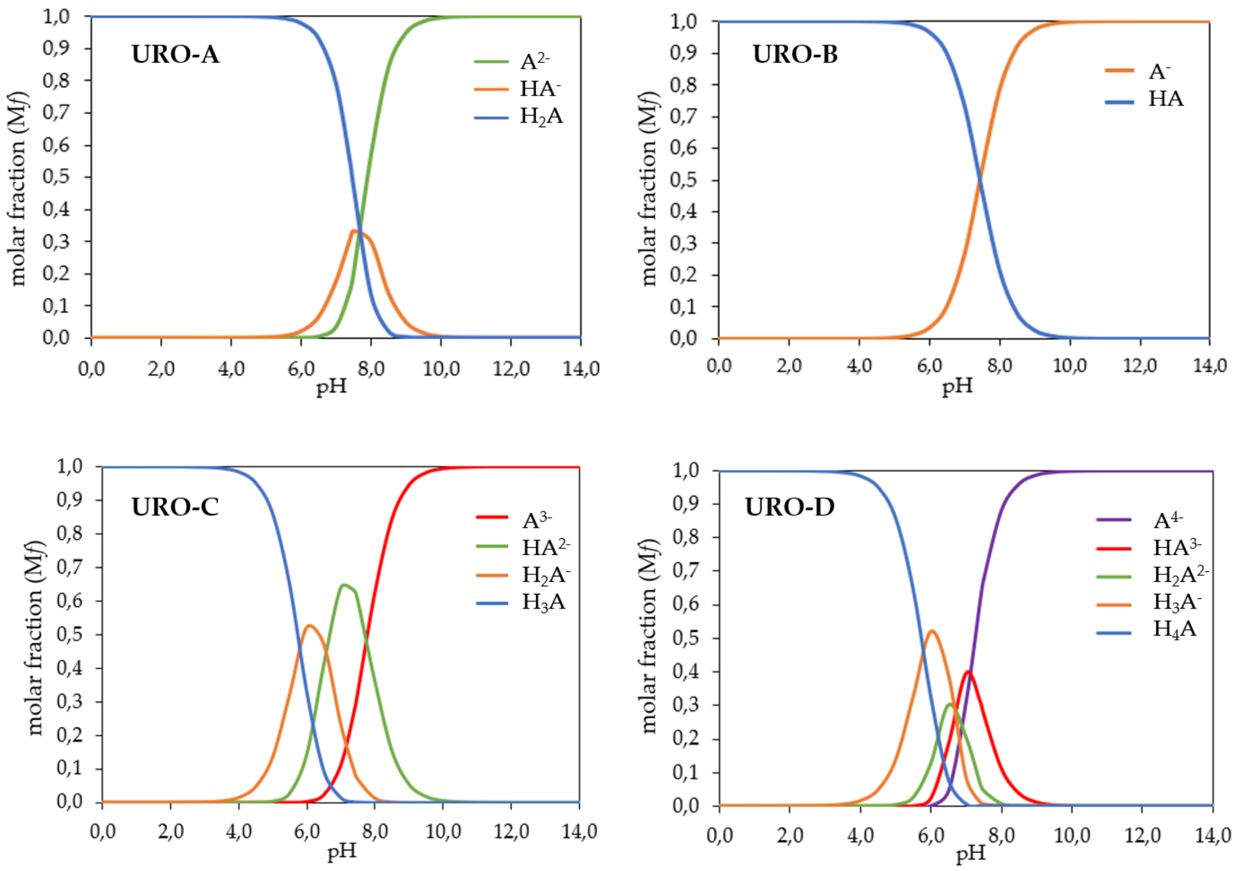

Figure S1. Distribution diagrams of UROs as function of pH.

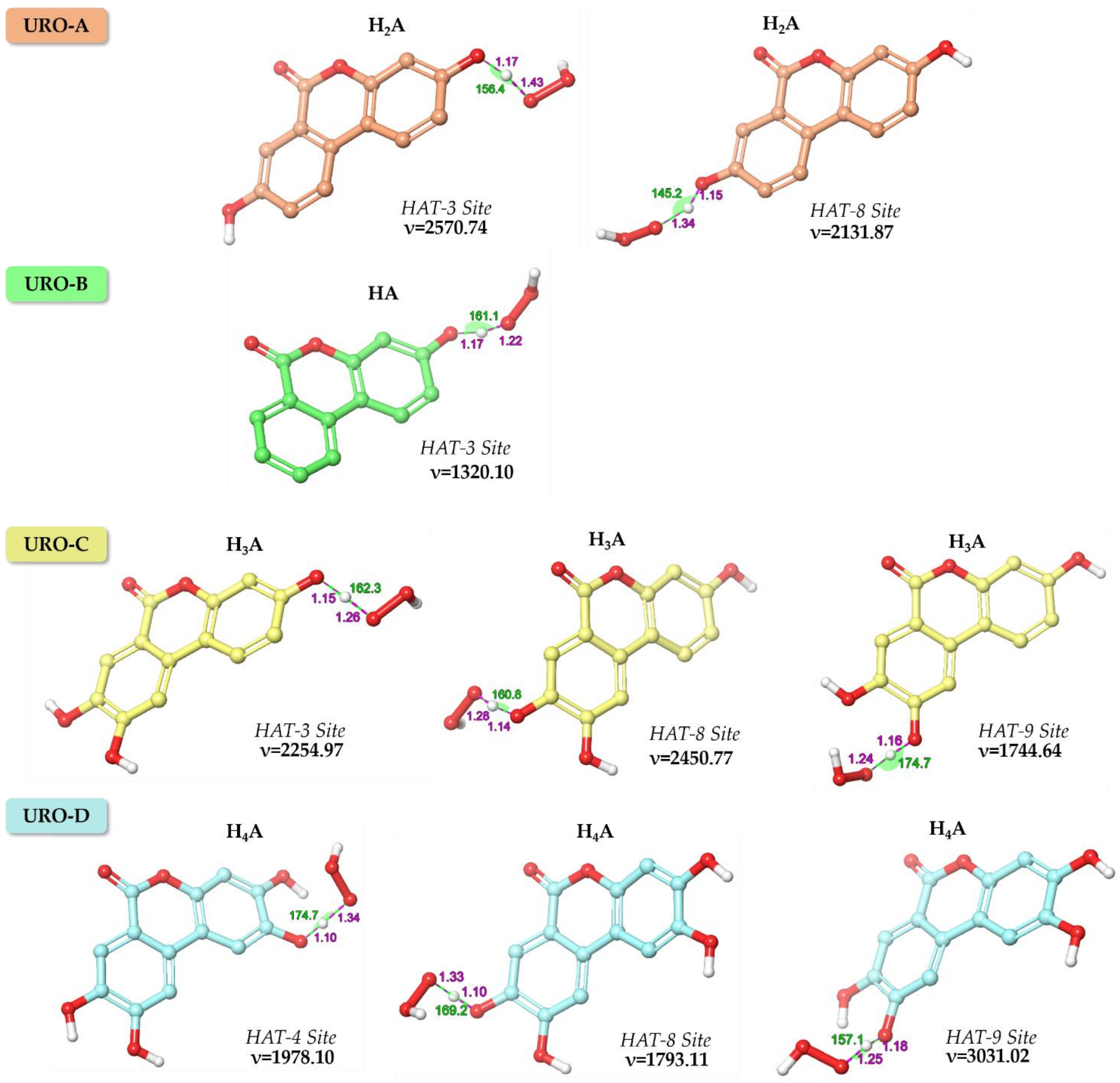

Figure S2. Geometries of the transition states of URO-A, URO-B, URO-C and URO-D obtained for the HAT reaction pathways in pentyl ethanoate (PE). Bond lengths (purple), angles (green) and imaginary frequencies (ν) are reported in Å, degrees and cm^{-1}, respectively.

Table S1. Nuclear organization term (λ) for the calculation of the barrier of reaction in SET mechanism, computed using the Marcus theory.

	**specie**	**ΔG°**	**ΔE**	$\boldsymbol{\lambda}$	**ΔG‡**
**URO-A**	HA^-	-10.83	29.83	40.66	5,47
**URO-B**	HA	-5.03	89.66	94.69	21.23
**URO-C**	H_2A^-	-7.70	34.61	42.31	7.08
	H_3A^-	-25.04	19.89	44,93	2.20
**URO-D**	HA^{3-}	-16.73	-4.43	12,30	0,40
	A^{4-}	-22.20	-10.38	11.82	2.28

Table S2. Rate Constants (k_{app}), expressed in M^{-1} s^{-1}, in pentyl ethanoate and branching ratios (Γ) computed at M05-2X level of theory at 298.15 K.

	**Mechanism**	**site**	k_{app}	**Γ(%)**
**URO-A**	HAT	*3*	1.72×10^{-10}	~0.00
	HAT	*8*	4.27×10^{-6}	100.00
**URO-B**	HAT	*3*	4.97×10^{-8}	100.00
**URO-C**	HAT	*3*	6.15×10^{-11}	0.04
	HAT	*4*	5.70×10^{-12}	~0.00
	HAT	*8*	1.48×10^{-7}	99.95
**URO-D**	HAT	*4*	6.30×10^{1}	96.06
	HAT	*8*	2.62×10^{-2}	0.04
	HAT	*9*	2.56×10^{1}	3.90

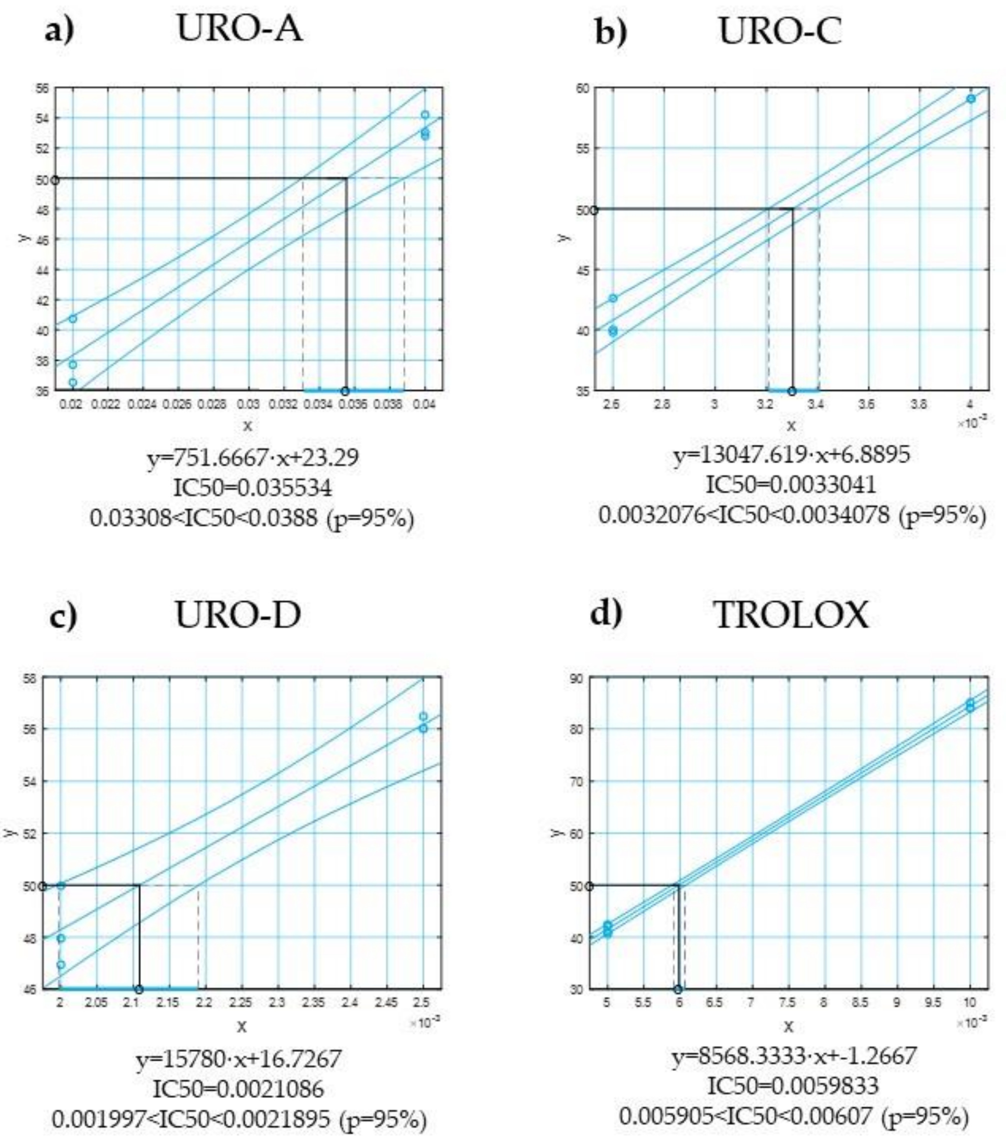

Figure S3. Regression lines for the calculation of the IC_{50} values.

a)

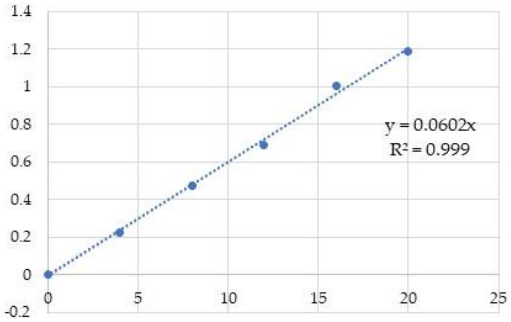

b)

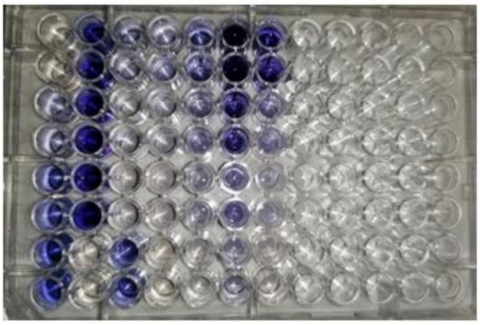

Figure S4. (**a**) Calibration curve of FRAP assay; (**b**) The microplate containing standards and samples at different concentrations.
